# The Effects of Exercise and Relaxation on Health and Wellbeing

**DOI:** 10.1002/hec.3477

**Published:** 2017-03-09

**Authors:** Hannah Forbes, Eleonora Fichera, Anne Rogers, Matt Sutton

**Affiliations:** ^1^ Manchester Centre for Health Economics University of Manchester Manchester UK; ^2^ NIHR CLAHRC Wessex, Faculty of Health Sciences University of Southampton Southampton UK

**Keywords:** self‐management, long‐term conditions, panel data, simultaneous recursive trivariate regression

## Abstract

Better management by individuals of their long‐term conditions is promoted to improve health and reduce healthcare expenditure. However, there is limited evidence on the determinants and consequences of self‐management activity. We investigate the determinants of two forms of self‐management, exercise and relaxation, and their impact on the health and wellbeing of 3472 individuals with long‐term health conditions over a 1‐year period. We use simultaneous recursive trivariate models to estimate the effects of these two inputs on three health and wellbeing outcomes: the EuroQol five‐dimensional (EQ‐5D) score, self‐assessed health and happiness. We reflect the opportunity cost of time and knowledge with employment status and education and find that employment reduces relaxation and education increases exercise. We find that neither exercise nor relaxation affects the EuroQol five‐dimensional score, but exercise increases self‐assessed health and relaxation increases happiness. Our findings show that individuals tailor their self‐management activities to their economic constraints, with effects on different aspects of their utility. Interventions to encourage self‐management should take account of heterogeneous effects and constraints. © 2017 The Authors. *Health Economics* Published by John Wiley & Sons, Ltd.

## 1 Introduction

Healthcare systems in Organisation for Economic Co‐operation and Development (OECD) countries are endeavouring to rise to the challenges emerging from ageing populations, growing levels of chronic illness, advancing technical possibilities and rising public expectations. These challenges have resulted in healthcare expenditure growing more rapidly than GDP in all OECD countries over the past 30 years (Pammolli *et al*., 2011). The downturn in the world economy has added to this and raised concerns regarding the sustainability of expenditure on healthcare systems (Tooke, 2011). In the UK, spending on the National Health Service (NHS) as a proportion of GDP is expected to fall from 8% to just over 6% by 2021 (Appleby *et al*., 2014). This could result in a funding gap of approximately £30 billion by 2020/21 (Illmann).

One way to reduce pressure on formal health services is to reduce demand, especially amongst people with long‐term conditions. According to Roberts *et al*. (2012), pressure on NHS costs from people with long‐term conditions is at least equal to, if not more than, that from the expanding and ageing population. It is estimated that there are more than 15 million people in the UK with at least one long‐term condition, and the number of people with multiple long‐term conditions has been predicted to rise by over a third over the next 10 years (Department of Health, 2012). There is a similar trend across OECD countries; in 1990, average spending on long‐term conditions was 0.7% of GDP, and this had risen to 1.5% of GDP by 2012 (Gershlick, 2015).

A sustainable way to manage demand on services used by people with long‐term conditions is to encourage people to engage in their own health and healthcare. This was described by Wanless (2002) as the ‘fully engaged scenario’. This vision has resulted in heightened policy emphasis on encouraging patients with long‐term conditions towards ‘self‐management’ through schemes such as the Expert Patients Programme (Department of Health, 2001). Self‐management has been defined by the Department of Health as ‘[t]he care taken by individuals towards their own health and wellbeing: it comprises of the actions they take to lead a healthy lifestyle; to meet their social, emotional and psychological needs; to take care of their long‐term condition; and to prevent further illness or accidents’ (Department of Health, 2005). There has been increasing advocacy of self‐management in the UK and internationally over the last two decades (Rogers *et al*., 2009).

Recently, Naylor *et al*. (2015) have argued that active self‐management support is one of the key priorities for healthcare commissioners to focus on helping to sustain the NHS. Self‐management support aims to increase ‘the capacity, confidence and efficacy of the individual’ to manage their condition (Kennedy *et al*., 2013). There are two core models of self‐management support: a provider‐based model with support embedded in clinical practice and a patient‐based model, which enables patients through individual or group‐based education, telehealth or telecare (Kennedy *et al*., 2013). Despite the growing policy emphasis on the importance of self‐management support, there is modest evidence of the effects on health and healthcare utilization (Griffiths *et al*., 2007; Kennedy *et al*., 2013; Richardson *et al*., 2008). More notably, evaluations of interventions to increase self‐management activity focus on self‐efficacy (the confidence of the individual to manage their condition through self‐management) and changes in health and healthcare utilization in the short term. Little attention has been paid to the self‐management activities *per se*, which are treated as intervening variables. In this paper, we aim to bridge the gap between self‐management literature and economic literature to enhance understanding of self‐management behaviour.

Previous studies have shown that the allocation of time to a health input such as self‐management depends on its associated opportunity cost. Brown and Roberts (2011) analysed the frequency of participation in physical activity by using six waves of data from Household Income and Labour Dynamics of Australia. They measured opportunity cost through employment status and found that full‐time employment had a significant negative impact on exercise for women. Mullahy and Robert (2010) hypothesized that the opportunity cost of time differs by level of human capital endowment (measured through education). Using data from the American Time Use Survey, they found that human capital endowments affect the way participants use their time to produce different health outcomes. For example, more educated individuals were more likely to participate in physical exercise. These studies indicate that differences in the cost of time can change the way that people choose to invest in self‐management.

Brown and Roberts (2011) and Mullahy and Robert (2010) both used general population data. One study that focuses on people with long‐term conditions is by Ettner *et al*. (2009). Unlike Mullahy and Robert (2010), Ettner *et al*. found that education *reduced* the amount of time spent on self‐management activities in a sample of 11 927 patients with at least one long‐term condition. This implies that time constraints may affect the choice of health inputs differently amongst people with long‐term conditions.

Changes in health inputs as a result of time constraints can also affect health outcomes (Balia and Jones, 2008; Contoyannis and Jones, 2004). Despite the clear link between inputs to health and health outcomes, we have found only one paper that investigates the link between opportunity cost and health through the choice of health inputs. Kuvaja‐Köllner *et al*. (2013) used 2 years of data from a randomized controlled trial in Finland to link the effect of the opportunity cost of time spent exercising with the effect of exercise on health. They assume physical exercise to be endogenous and so conduct a two‐stage least squares analysis, using motivation to exercise as the instrumental variable for exercise. Kuvuja‐Köllner *et al*. find that a lower opportunity cost of time (measured through labour market status) has a significant effect on the amount of time spent exercising. This increase in time spent on physical exercise was found to have a positive effect on the short form 6D.

The Kuvaja‐Köllner *et al.* (2013) paper shows that the opportunity cost of time affects health inputs and health outcomes. However, their instrumental variable is a short‐term decision that could be correlated with the outcome. It could be that enjoyment of physical exercise can change and therefore is not a suitable instrument for exercise. A further limitation of Kuvaja‐Köllner *et al*.'s (2013) analysis is that lagged measures of health are not included in the model. Previous endowments of health are likely to affect people's allocation to time spent on exercise as well as people's future health. Therefore, the results could be affected by omitted variable bias. In addition, the input (exercise) and output (health) equations were not modelled simultaneously, even though it is likely that the errors between these equations could be correlated.

Kuvaja‐Köllner *et al*. (2013) paved the way for linking a health input to a health output within the same dataset, but they did not explain how the opportunity cost of time could affect the specific self‐management activities that policy is aiming to encourage. In this paper, we describe how the opportunity cost of time (measured through employment and education) can impact on the mix of self‐management activities and estimate the effects of self‐management activities on future health and wellbeing. Our study is distinct because we study a population of individuals with long‐term conditions, precisely those for whom self‐management potential is greatest. As we use data from a clinical trial, we have rich data on health and wellbeing. We improve on Kuvaja‐Köllner *et al*.'s (2013) study by modelling inputs and outputs simultaneously and by using more plausible identifying restrictions. We estimate exercise, relaxation and health as part of a simultaneous model and allow for the possibility that there is correlation between these equations.

## 2 Methods

### 2.1 Theoretical motivation

We motivate our empirical estimation of the relationship between the opportunity cost of time and self‐management (measured through exercise and relaxation) with a modified time allocation framework. The time allocation framework is based upon Becker's (1965) theory of allocation of time and the Cawley (2004) ‘SLOTH’ (sleep, leisure, occupation, transportation and home‐based activities) framework. We can think of patients who demand health for two reasons: (i) as a consumption commodity, because it directly enters their preference functions, and (ii) as an investment commodity, because it determines the amount of time available for other activities. Individuals choose how to allocate their time amongst work, exercise, relaxation and other activities to maximize their utility subject to a budget and time constraint. The budget constraint includes a time price of ‘self‐management’ which is dependent on wages and educational qualifications. The time price indicates the opportunity cost of participating in exercise and relaxation. The budget constraint includes the money price of exercise (e.g. gym membership), income and the time price of working. Health output is determined by two inputs, exercise and relaxation. If the time price of exercise increases due to the individual entering employment, then the opportunity cost of time spent exercising increases. But as the individual becomes richer, he or she is able to afford the money price of exercise. The overall effect of a change in the time price of exercise on its demand is ambiguous and depends on the relative size of the income and substitution effects.

### 2.2 Data

We use data collected in the trial of Whole System Informing Self‐Management Engagement, a provider‐based model of self‐management support (Bower *et al*., 2012; Kennedy *et al*., 2014, 2013). The trial was based in the north‐west of England between the years of 2009 and 2012. The population in the local area was predominantly white and socioeconomically deprived. The objective of the trial was to determine the effectiveness of a provider‐based model of self‐management support. No statistically significant differences were found between the patients receiving the provider‐based intervention and those in the control group. However, the trial highlighted the need to elicit and understand the active components required for effective self‐management support (Kennedy *et al*., 2014).

Patients were eligible for the trial if they had any of three long‐term conditions: diabetes, chronic obstructive pulmonary disease and irritable bowel syndrome. These were selected as exemplar conditions that could be influenced by self‐management interventions and where there was also published evidence of effectiveness (Deakin *et al*., 2005; Richardson *et al*., 2006). Patients were identified from electronic health records and checked for any exclusion criteria (under 18 years of age, insufficient English language, receiving palliative care or insufficient capacity to give written consent) before being sent a postal invitation to take part in the trial. Forty‐four general practices were recruited.

#### 2.2.1 Health variables

We consider three measures of health and wellbeing: the EuroQol five‐dimensional three‐level (EQ‐5D‐3L) score, self‐assessed health (SAH) and the amount of time the respondent had spent being happy over the previous 4 weeks. The five dimensions in the EQ‐5D are mobility, self‐care, usual activities, pain/discomfort and anxiety/depression. Each dimension provides three options of response: no problems, some problems and extreme problems. SAH was collected by asking the following question: ‘In general, would you say your health is excellent, very good, good, fair or poor?’ Happiness was collected by using the following question: ‘How much time during the past 4 weeks have you been a happy person?’ The individual was offered six response categories: none of the time, a little bit of the time, some of the time, a good bit of the time, most of the time and all of the time.

#### 2.2.2 Measures of self‐management (health inputs)

The respondents were asked to report the self‐management activities they adopted to help manage their conditions through the question: ‘To help manage my condition, I…’ For each listed self‐management activity, the respondent could select whether it was very true, partly true or not true. A full list of self‐management activities and associated summary statistics is presented in Table 2. Amongst the 12 listed activities are ‘do exercises’ and ‘rest and relax’. We focus on these inputs because both activities are time consuming and therefore economically interesting. They are also not highly correlated to one another (*ρ* = 0.07) and demonstrate good variation in the sample. In preliminary analysis, we also studied responses to ‘I follow an eating plan or special diet’, but we did not find any significant effects on the outcome variables.

#### 2.2.3 Education and economic status

The participants were asked to report their highest educational qualification. We created a dummy variable for higher education. A value of 0 captured those with no qualifications, O levels or A levels, which constituted 51.3% of the participants. A value of 1 represents those with a professional qualification (36.7%) or a degree (12.0%).

The respondents were also asked to report their employment status from a list of eight options. We created a dummy variable. A value of 0 captured those who were unemployed, retired from paid work, voluntary workers and others. This comprised 65.1% of participants. A value of 1 represented those who were in full‐time or part‐time work, self‐employed, looking after the family or home or in full‐time education, capturing the other 34.9% of the participants. We combined the categories in this way because we found the effects of being in full‐time education and looking after the family or home to be very similar to the effects of full‐time work. They are equally as influential intuitively in terms of time availability.

#### 2.2.4 Covariates

To control for the previous health of the participants, we used the respondent's SAH score and EQ‐5D‐3L score at baseline and at 6 months, as well as the time spent being happy at 6 months. We also controlled for socioeconomic and demographic characteristics, including gender, age, ethnicity, type of accommodation (defined as dummy variable with a value of 1 indicating whether the respondent owns a house) and type of long‐term condition. We further controlled for deprivation by using the index of multiple deprivation score [Index of Multiple Deprivation (IMD), 2007] of the respondent's area of residence.

#### 2.2.5 Analysis sample

The data were collected from patients via postal questionnaires at baseline and 6 and 12 months. We use all three waves of the data. Initially, 5599 patients (2546 with diabetes, 1634 chronic obstructive pulmonary disease and 1419 with irritable bowel syndrome) were recruited to the trial. Four thousand five hundred thirty‐three patients (81.0%) completed the 6‐month follow‐up questionnaire, and 4076 (72.8%) completed the 12‐month questionnaire.

We follow the missing data strategy adopted by Kennedy *et al*. (2013) who collected the dataset. This strategy was based on the findings of a simulation study by Groenwold *et al*. (2012), who showed that covariate adjustment is as efficient as multiple imputation at controlling for bias with the advantage of improving precision in estimating the treatment effect. We did not impute missing values for the outcome variables at 6 and 12 months but addressed potential bias due to missingness with covariate adjustment. We used logistic regression to model missingness in the outcome variables as a function of demographic characteristics, employment status, ethnicity, type of health condition, type of accommodation, the index of multiple deprivation, EQ‐5D‐3L and SAH measured at baseline. We then controlled for the baseline covariates that were predictive of missingness at the two follow‐ups.

There were small numbers (less than 3% of the sample) of missing values in the baseline values of EQ‐5D‐3L, SAH, gender, age, ethnicity, type of housing tenure and diabetes, heart problems/blood pressure and anxiety/depression. Missing values for these baseline variables were imputed by using a multiple imputation through chained‐equation technique as in Kennedy *et al*. (2013). Three thousand four hundred and seventy‐two observations were available following the imputation of missing data on the baseline variables.

### 2.3 Empirical strategy

Our empirical strategy aims to investigate the determinants of self‐management activities and the effect of these health inputs on health outputs. Self‐management activities such as exercise and relaxation have the potential to be endogenous if there is an unobserved variable that is correlated with both these inputs and health outputs or there could be unobserved individual characteristics that drive both the extent of self‐management activity and health. This potential for endogeneity raises the concern that if we estimate these equations separately, then there would be unobserved correlation between the error terms. Therefore, we adopt a simultaneous recursive trivariate model that controls for the potential endogeneity of exercise and relaxation and for unobserved heterogeneity between the equations (Maddala, 1983). Our model simultaneously estimates three equations, one output equation (health) and two input equations (exercise and relaxation):

(1) $$h_{t}=\;\alpha_{1}+\beta_{1}h_{0}+\delta_{1}h_{t-1}+\gamma_{1}x_{t}\;+\mu_{1}e_{t}+\sigma_{1}r_{t}\;+\;u_{1t}$$

(2) $$e_{t}=\alpha_{2}+\beta_{2}h_{0}+\delta_{2}h_{t-1}+\gamma_{2}x_{t}\;+\phi_{2}e_{t-1}+\omega_{2}r_{t-1}+\;\vartheta_{2}w_{0}+\tau_{2}ed_{0}+u_{2t}$$

(3) $$r_{t}=\alpha_{3}+\;\beta_{3}h_{0}+\delta_{3}h_{t-1}+\gamma_{3}x_{t}\;+\phi_{3}e_{t-1}+\omega_{3}r_{t-1}+\;\vartheta_{3}\;w_{0}+\tau_{3}ed_{0}\;+\;u_{3t}$$

Health at baseline and 6 months is denoted by *h*_0_ and *h*_t − 1_, and *x* denotes sociodemographic variables. Equations 2 and 3 show that we exclude employment (*w*_0_) and education (*ed*_0_) from the health equation. Thus, we assume that, conditional on baseline and lagged health and the other covariates, employment and education affect health indirectly through their influence on exercise and relaxation.

The variance–covariance matrix Ω can be written as follows:

$$\Omega =\left(\begin{array}{ccc} 1 & \rho_{12} & \rho_{13}\\ \rho_{12} & 1 & \rho_{23}\\ \rho_{13} & \rho_{23} & 1 \end{array}\right)$$

where *ρ*_jk_ with *j*, *k* = 1, 2,3 ∀ *j* ≠ *k* is the correlation between the error terms. Unobserved heterogeneity can be allowed with the potentially non‐zero values of the off‐diagonal elements of the matrix. We use Roodman's (2011) conditional recursive mixed process model to estimate the system of equations (1–3) in stata and report them in Table 4.

There has been much discussion of the best estimation technique for EQ‐5D given its bimodal distribution. Hernández Alava *et al*. (2012) argue that estimation of EQ‐5D should take into account its bimodal distribution by using an adjusted limited dependent variable mixture model. Other researchers have also used two‐part Tobit models (Acaster *et al*., 2015). However, numerous studies find that when EQ‐5D is treated as continuous, the model performs just as efficiently as any other estimation strategy, if not better (Kim *et al*., 2014).

To check for robustness of our results, we also run our analysis treating SAH and happiness as ordered probits to reflect the ordinal nature of these dependent variables. We present average marginal effects for each possible response category (none of the time, a little of the time, etc.).

Exercise and relaxation are ordinal variables on a three‐point scale. We include these variables in two different ways. When we model the health/wellbeing variables using linear models, we also treat the self‐management inputs as continuous. When we model SAH and happiness as ordinal variables, we include the self‐management input as binary variables, separating those who reported ‘very true’ from those who reported ‘partly true’ or ‘not true’.

We also test the independence of the equations and the validity of the over‐identifying restrictions. We test the independence of the equations with a likelihood ratio test that compares an unrestricted equation with all correlations between equations and a restricted one with correlations set to zero. We conduct an *F*‐test for over‐identification by setting the coefficients of the instruments equal to 0, excluding lagged exercise for exercise and lagged relaxation for relaxation.

## 3 Results

### 3.1 Descriptive statistics

At baseline, the average EQ‐5D score reported by the respondents was 0.655, ranging from −0.426 to 1 (Table I). Also at baseline, 40% of the respondents reported their SAH as ‘poor’ or ‘fair’, and 43% stated that they were happy ‘none of the time’, ‘a little bit of the time’ or ‘some of the time’.

**Table I:** Descriptive statistics

Variable	At baseline	At 6 months	At 12 months
EQ‐5D score (mean, SD)	0.655 (0.307)	0.637 (0.312)	0.636 (0.317)
Self‐assessed health (*n*, %)			
Poor	302 (8.70%)	380 (10.94%)	405 (11.66%)
Fair	1108 (31.91%)	1044(30.07%)	1082 (31.16%)
Good	1365 (39.31%)	1266 (36.46%)	1238 (35.66%)
Very good	607 (17.48%)	688 (19.82%)	636 (18.32%)
Excellent	90 (2.59%)	94 (2.71%)	111 (3.20%)
Happiness (*n*, %)			
None of the time	Not available	238 (6.85%)	218 (6.28%)
A little bit of the time		493 (14.20%)	475 (13.68%)
Some of the time		761 (21.92%)	836 (24.08%)
A good bit of the time		656 (18.89%)	639 (18.40%)
Most of the time		1022 (29.44%)	1002 (28.86%)
All of the time		302 (8.70%)	302 (8.70%)
Higher education (*n*, %)	1688 (48.62%)		
Working full time (*n*, %)	1158 (33.35%)		
White ethnic background (*n*, %)	3387 (97.55%)		
Owns accommodation (*n*, %)	2443 (70.36)		
Long‐term condition (*n*, %)			
Diabetes	1736 (50.00%)		
Chronic obstructive pulmonary disease	1247 (35.92%)		
Irritable bowel syndrome	1014 (29.21%)		
Arthritis	1586 (45.68%)		
Heart	1547 (44.56%)		
Fatigue	121 (3.49%)		
Anxiety	678 (19.53%)		
Multiple sclerosis	13 (0.37%)		
Other	686 (19.76%)		
Number of individuals	3472		

EQ‐5D score, EuroQol five‐dimensional score.

The average age of the participants was 63 years (ranging from 18 to 102 years). The sample was predominantly from a White ethnic group (97%), slightly higher than the 95% across England as a whole for the population aged 65 years (see Office for National Statistics, 2009) and reflecting the population composition of Salford. A large proportion of the sample (71%) owned their own accommodation. Diabetes is the most common long‐term condition of the participants, with 50% suffering the condition. Arthritis and heart conditions were the next most frequent at 46% and 45% respectively.

At 12 months, 30.2% of the respondents said that it was ‘not true’ that they used exercise to manage their condition, 46.6% said that it was ‘partly true’, and 23.2% said that it was ‘very true’ (Table II). Of the respondents, 9.5% said that it was ‘not true’ that they rested and relaxed, 47.6% said that it was ‘partly true’, and 42.3% said that it was ‘very true’. The descriptive statistics for the 10 other self‐management activities are also provided in Table II. We investigated the correlations among the 12 self‐management activities and found that exercise was most strongly correlated with diet (*ρ* = 0.24) and relaxation was most strongly correlated with stress management (*ρ* = 0.39) and medication (*ρ* = 0.20). Exercise and relaxation were not highly correlated with each other (*ρ* = 0.07).

**Table II:** Descriptive statistics for all 12 self‐management activities in the questionnaire

Variable	Description	At 6 months	At 12 months
Exercise			
Not true	Do exercises	1062 (30.59%)	1047 (30.16%)
Partly true		1563 (45.02%)	1618 (46.60%)
Very true		847 (24.40%)	807 (23.24%)
Relaxation			
Not true	Rest and relax	341 (9.82%)	330 (9.50%)
Partly true		1638 (47.18%)	1653 (47.61%)
Very true		1493 (43.00%)	1489 (42.89%)
Diet			
Not true	Follow an eating plan or special diet	1064 (29.09)	1043 (28.55%)
Partly true		1762 (48.17%)	1833 (50.18%)
Very true		832 (22.74%)	777 (21/27%)
Stress management			
Not true	Reduce or avoid stress	407 (11.07%)	409 (11.12%)
Partly true		1863 (50.65%)	1851 (50.31%)
Very true		1408 (38.28%)	1419 (38.57%)
Medication			
Not true	Take medication as prescribed	126 (3.44%)	134 (3.66%)
Partly true		162 (7.16%)	277 (6.57%)
Very true		3271 (89.40%)	3249 (88.77%)
Information			
Not true	Seek information about condition	605 (16.48%)	633 (17.27%)
Partly true		1528 (41.62%)	1506 (41.09%)
Very true		1538 (41.90%)	1526 (41.64%)
Alternative treatments			
Not true	Try alternative treatments such as health products	2694 (73.49%)	2722 (74.39%)
Partly true		557 (15.19%)	547 (14.95%)
Very true		415 (24.40%)	390 (10.66%)
Meditation			
Not true	Meditation or prayers	2896 (79.32%)	2892 (79.43%)
Partly true		452 (12.38%)	434 (11.92%)
Very true		303 (8.30%)	315 (8.65%)
Advice from family/friends			
Not true	Seek advice from friends or family	2098 (57.14%)	2090 (57.15%)
Partly true		1248 (33.99%)	1251 (34.21%)
Very true		326 (8.88%)	316 (8.64%)
Advice from patients			
Not true	Seek advice from someone with the same condition	2382 (64.96%)	2379 (65.11%)
Partly true		990 (27.00%)	1008 (27.59%)
Very true		295 (8.04%)	267 (7.31%)
Self‐help groups			
Not true	Member of self‐help or support group	3449 (94.36%)	3430 (94.05%)
Partly true		92 (2.52%)	98 (2.69%)
Very true		114 (3.12%)	119 (3.26%)
Living adjustments			
Not true	Make adjustments to living conditions	1078 (29.35%)	1084 (29.55%)
Partly true			
Very true		1799 (48.98%)	1801 (49.10%)
		796 (21.67%)	783 (21.35%)
Number of individuals	3472		

### 3.2 Simultaneous trivariate recursive regression results

Being in full‐time employment decreases the probability that the respondent uses relaxation to manage their condition by 11 percentage points (Table III). Table III also shows that having a degree or other professional qualification increases the probability that the individual will use exercise to manage their condition by 5 percentage points respectively.

**Table III:** Simultaneous recursive estimation of the effects of health investments on health and wellbeing

	Outcomes at 12 months		
	EQ‐5D score	Self‐assessed health	Time a happy person
Outcome equation			
Exercise at 12 months	0.0018	0.0770***	0.0396
	(0.24)	(3.42)	(0.84)
Relaxation at 12 months	0.0074	−0.0423	0.2159***
	(0.63)	(−1.22)	(2.95)
Input equation (exercise at 12 months)			
Exercise at 6 months	0.5797***	0.5798***	0.5799***
	(42.49)	(42.51)	(42.52)
Relaxation at 6 months	0.0141	0.0146	0.0147
	(0.90)	(0.93)	(0.93)
Working full time	−0.0231	−0.0215	−0.0208
	(−0.86)	(−0.80)	(−0.77)
Higher education	0.0546**	0.0548**	0.0551**
	(2.67)	(2.68)	(2.70)
Input equation ^(^relaxation at 12 months)			
Exercise at 6 months	0.0110	0.0114	0.0110
	(0.82)	(0.85)	(0.82)
Relaxation at 6 months	0.4150***	0.4152***	0.4150***
	(26.86)	(26.89)	(26.86)
Working full time	−0.1108***	−0.1121***	−0.1105***
	(−4.18)	(−4.24)	(−4.17)
Higher education	−0.0356	−0.0355	−0.0354
	(−1.77)	(−1.77)	(−1.76)
*ρ*_12_	0.0500	−0.0043	−0.0245
	(1.75)	(−0.15)	(−0.86)
*ρ*_13_	−0.0109	0.0588	−0.0200
	(−0.28)	(1.55)	(−0.52)
*ρ*_23_	0.0819***	0.0819***	0.0819***
	(4.78)	(4.78)	(4.78)
No. of observations	3472	3472	3472

EQ‐5D score, EuroQol five‐dimensional score.

Exercise and relaxation are measured as continuous variables: 0 = ‘not true’, 1 = ‘partly true’ and 2 = ‘very true’. All models are estimated on five datasets with imputed values for the missing variables. All equations also include the following covariates: ethnicity, housing tenure, index of multiple deprivation, main health condition, age, age squared, baseline self‐assessed health, self‐assessed health at 6 months, happiness at 6 months, EQ‐5D at baseline and EQ‐5D at 6 months.

*** *p* < 0.001.

** *p* < 0.05. *t*‐stats are in parentheses.

Exercise and relaxation are found to have no statistically significant effect on EQ‐5D. A one‐unit increase in exercise, which represents a change from ‘not true’ to ‘partly true’ or ‘partly true’ to ‘very true’ that this form of self‐management was used by the respondent, increases SAH by 0.08 units on a five‐point scale. The same change in the use of rest and relaxation increases happiness by 0.22 units on a six‐point scale.

The value for *ρ*_23_ is positive and significant (Table III). This implies that those who have a higher propensity to exercise also have a higher propensity to relax (and vice versa).

### 3.3 Robustness checks

The results of the simultaneous recursive trivariate models where SAH and happiness are treated as ordinal variables are presented in Table IV. Exercise increases the probability that SAH is good, very good or excellent and reduces the probability that it is either poor or fair. Relaxation instead decreases the probability that SAH is good, very good or excellent and increases the probability that it is either poor or fair. Relaxation significantly increases the probability of being happy a good bit of the time, most or all of the time.

**Table IV:** Marginal effects of exercise and relaxation on self‐assessed health (SAH) and happiness

	Self‐assessed health					Happiness					
	Poor	Fair	Good	Very good	Excellent	None of the time	A little of the time	Some of the time	A good bit of the time	Most of the time	All of the time
Exercise	−0.025***	−0.022***	0.007*	0.029***	0.011***	−0.007	−0.007	−0.006	0.001	0.011	0.008
Relaxation	0.018	0.016	−0.005	−0.021	−0.008	−0.054*	−0.059**	−0.047*	0.006*	0.088*	0.066*

Estimated with simultaneous trivariate recursive equations treating SAH and happiness as ordered probits and exercise and relaxation as binary variables, 1 = ‘very true’, 0 = ‘partly true’ or ‘not true’. All equations also include the following covariates: ethnicity, housing tenure, index of multiple deprivation, main health condition, age, age squared, baseline self‐assessed health, self‐assessed health at 6 months, happiness at 6 months, EuroQol five‐dimensional score (EQ‐5D) at baseline and EQ‐5D at 6 months.

*** *p* < 0.001.

** *p* < 0.01.

* *p* < 0.05.

### 3.4 Test statistics

The test statistics in Table V show that the equations are not independent. The *p*‐values from a likelihood ratio test imply that we should reject the null hypothesis and conduct a joint analysis. The results of an *F*‐test for over‐identification indicate that we can reject the null hypothesis; the model is not over‐identified.

**Table V:** Tests of over‐identification and residual independence in the simultaneous recursive trivariate model

	Health/wellbeing outcome		
Test	EQ‐5D score	Self‐assessed health	Happiness
Over‐identification	*χ* ^2^ (2) = 31.43	*χ* ^2^ (2) = 32.86	*χ* ^2^ (2) = 31.44
	Prob > *χ* ^2^ < 0.0001	Prob > *χ* ^2^ < 0.0001	Prob > *χ* ^2^ < 0.0001
Independence of residuals	*χ* ^2^ (3) = 26.06	*χ* ^2^ (3) = 25.30	*χ* ^2^ (3) = 23.79
	Prob > *χ* ^2^ < 0.0001	Prob > *χ* ^2^ < 0.0001	Prob > *χ* ^2^ < 0.0001

EQ‐5D score, EuroQol five‐dimensional score.

## 4 Discussion

In this paper, we have examined the determinants and consequences of two self‐management activities, relaxation and exercise. We have motivated our empirical specification with a time allocation framework where a change in the opportunity cost of time has an ambiguous effect on the choice of these health inputs because of the relative size of the income and substitution effects. As a result, the effect of exercise and relaxation on health is ambiguous as well. We apply this framework to a unique longitudinal trial of 3472 individuals observed at three points in time. This group is representative of populations with long‐term conditions, exactly those at whom self‐management support schemes are targeted.

We use education and employment status as measures of the opportunity cost of time of health investments. If the substitution effect is dominant, then increases in the opportunity cost of time (being in full‐time employment or having a degree) should reduce the amount of time participating in these activities. An increase in the opportunity cost of time also means an increase in income (if you are working or have higher education, then you are likely to have a higher income). Individuals are more likely to trade off labour for leisure as real income increases as long as leisure is a normal good.

Our results indicate that those who work full time are less likely to spend time resting and relaxing as a form of self‐management compared with those who do not work full time. This implies that the substitution effect is dominating. People are substituting self‐management for working as the opportunity cost of self‐management is increasing. This finding coincides with Humphrey and Ruseski's (2011) paper.

Education increases the probability of exercising. This implies that the income effect is dominating (when the shadow price of time is higher, the respondents have higher income and therefore do not need to spend as much time working). This finding is consistent with research on exercise within the general population (Eberth and Smith, 2010).

Time preference has been suggested as a channel through which health and education are correlated (Grossman, 2006). Van der Pol (2011) found that the relationship between education and health can be partly explained through time preference. Our finding that education increases exercise and decreases relaxation could also be partly explained by time preference. People with higher education could have a lower time preference and therefore are more willing to engage in an activity that benefits health in the long term, whereas people with lower education might prefer to engage in a self‐management that has a more instantaneous payoff.

Employment status and education change the way that people allocate their time to health investments. This finding supports similar findings in the literature (Brown and Roberts, 2011; Kuvaja‐Köllner *et al*., 2013; Mullahy and Robert, 2010). We find that the way in which people decide to allocate their time to self‐management behaviours affects their health. For example, as found by Kuvaja‐Köllner *et al*. (2013), exercise has a positive effect on SAH. Our findings could explain why researchers have found self‐management support schemes to affect efficacy but to only have modest effects on health and patient outcomes. One way to encourage people to engage in their health could be to attempt to make health‐promoting behaviours less costly to the individual.

One of our key findings is that the effect of exercise and relaxation on health and wellbeing depended on which measures of health and wellbeing we used. We found that both self‐management activities had no significant effect on EQ‐5D score, whereas exercise had a positive effect on SAH and relaxation had a positive effect on happiness. This is likely to be because different measures capture different aspects of health and wellbeing. For example, Sun *et al*. (2016) found that the anxiety/depression domain of the EQ‐5D was most strongly associated with subjective wellbeing. Böckerman *et al*. (2011) found that psychiatric disorders had a large effect on wellbeing even after controlling for the EQ‐5D (Böckerman *et al*., 2011). This could explain the stronger relationship shown between relaxation and happiness, due to its conceivably greater effect on mental health.

This may also be the explanation for the positive effect of exercise on SAH, which may be more focused on physical health. Exercise may positively affect physical health more than any other aspect, and hence show up most significantly in SAH. The lack of significant effect of either exercise or relaxation on EQ‐5D scores over time, however, may be explained by its insensitivity to changes over a short period of time. Clearly, the potential differences among EQ‐5D, SAH and happiness could have important ramifications for policy and allocation of resources depending on what measure we take to best capture utility.

Our test statistics indicate that there is enough correlation between the errors to justify modelling the equations simultaneously. Through modelling the equations simultaneously, we are also controlling for two sources of unobserved heterogeneity. First, we control for the possibility that exercise and relaxation are endogenous. We also control for the possibility that there is unobservable heterogeneity between the equations in our model. Through conducting a joint analysis, we found that the propensity to participate in relaxation is positively correlated with the propensity to participate in exercise. This could be picking up a measure of unobserved motivation to engage in health‐promoting behaviour.

To our knowledge, this is the first study in the UK that links the effect of employment and education on health inputs and health. We are also the first study to our knowledge to model exercise, relaxation and health simultaneously. However, there are several limitations to this study. First, there is a relatively short follow‐up. We look at the effect that self‐management has on health after just 6 months. One explanation for our modest findings for the effect of self‐management on EQ‐5D could be that 6 months is too short to pick up most of the effects of self‐management behaviour. Another potential limitation of the study is that we assume that exercise and relaxation are separate inputs with additive effects. We also note that our results are confined to a group of older people with an ethnic composition that is not representative of the younger population in England.

A further limitation relates to the available measures of exercise and relaxation. A change from ‘partly true’ to ‘very true’ is not an easily transferable measure of health input. However, further analysis (Table IV) shows that our results do not differ when using binary variables for whether the respondents reported ‘very true’ versus ‘partly true’ or ‘not true’.

Overall, our study demonstrates that constraints on time can change the way individuals engage in self‐management, with effects on health and wellbeing. We find no effect of exercise and relaxation on EQ‐5D, but we find significant positive effects of exercise on SAH and relaxation on happiness. These results are relevant for the National Institute for Health and Clinical Excellence guidelines on best practice management of long‐term conditions (NICE Guidelines, 2009; NICE Guidelines, 2015). These guidelines promote a combination of drug treatment and behaviour change interventions targeting exercise but do not mention the importance of relaxation. Our findings indicate that initiatives designed to encourage self‐management should take account of heterogeneous constraints on an individual's potential to use different inputs and the potential for different effects on alternative aspects of their health and wellbeing. These results support the provision of individualized care and shared decisions between doctors and patients on the best behaviour change interventions depending on the individual's time constraints (NICE Guidelines, 2015).
